# Report of Diseases in the Public and Private Practice of One of the Physicians of the Finsbury Dispensary, from the 20th of May, to the 20th of June

**Published:** 1805-07-01

**Authors:** J. Reid

**Affiliations:** Greville Street, Brunswick Square


*Report of Diseases in the public and private Practice of one of the Physicians of the Finsbury Dispensary, from the 20th of May, to the 20th of June.*

| | | | |
|---|---|---|---|
| Amaurosis | 1 | Hysteria | 2 |
| Paralysis | 4 | Hypochondriasis | 4 |
| Insania | 1 | Epilepsia | 1 |
| Rheumatismus | 23 | Chorea sancti vitæ | 1 |
| Catarrhus | 11 | Colica Pictonum | 2 |
| Febricula | 7 | Hydrops | 6 |
| Amenorrhœa | 13 | Asthenia | 15 |
| Menorrhagia | 5 | Morbi Infantiles | 19 |
| Leucorrhœa | 3 | Morbi Cutanei | 17 |

In consequence of the suggestion of a professional friend, in whose judgment, as well as candour, he has an entire confidence, the Reporter is induced, before he makes any remarks upon the diseases of the last month, to explain a proposition which was made by him in the preceding number of this Journal, with regard to the cure and management of fever.

The writer of that article did not mean to say, that in all cases, or in the greater number, when a patient dies of fever, it must inevitably be owing to an error of the medical attendant. He wished merely to express his firm conviction, that, where the excitability had not been previously exhausted by years, or by habits of intemperance or indiscretion, a person who, from the *first feeling* of a febrile attack, was properly managed and attended to, (which, by the way, depends as much on the patient himself, his friends, or his nurse, as on his physician) would never fall a victim to the complaint.

When

When the assertor of this opinion shall meet, in his practice, with a fact that opposes it, he pledges himself to recant his faith, and to publish, without any colouring or mutilation, the actual circumstances of the case.

The fatal error consists in hesitating to apply for any advice, until that period of a disease when the best advice is often of no avail. "For want of timely care, millions have died of medicable wounds."

A remarkable instance of dimness of sight, that has for some months past been gradually approaching towards absolute blindness, which indeed has actually taken place in one of the eyes, is at present under the Reporter's care. The patient first perceived it the day after she had been frightened by a violent paroxysm of epilepsy with which her husband was attacked in the night. Since that time, although never in the least so before, she has herself become extremely liable to fits, and is apt to fall down insensible, upon occasions of the slightest degree of surprise or agitation. Her complaint seems to consist not in an injured structure of the eye, but in a debility of the nervous system in general, that *appears* more particularly in that delicate and exquisitely-irritable part of it which is destined for the purpose of vision. It is, of course, not a case for *surgical,* but *medical,* treatment*.

She already begins to feel an alleviation of her complaint. She says, that "she can bear the light of the sun better." Before, she was scarcely able to see at all, unless when in the house, or in some other way sheltered from the glare of the solar rays. The capacity of seeing with the eye that is not altogether blind is intermittent, "going and coming," to use her own metaphor, "like the sun when a cloud passes over it." The patient has likewise been liable to a deafness, that may be traced to the same circumstance as gave rise to her ophthalmic malady. Her hearing is in a great measure restored, which affords an agreeable

---

* Such distinctions, although for the most part they ought to be observed, may, sometimes, be carried to an absurd and ludicrous extent. At Calais, about three years ago, as the writer was walking upon the sands of the shore, a particle from them lodged itself under his upper eye-lid. Happening, at the moment of the accident, to be near a shop that wore a pharmaceutical physiognomy, he entered, and requested the person whom he saw there to remove the troublesome intruder, by means of the feather of a quill that was lying upon his desk. The performance of this operation, however, he scrupulously declined. Je vous demande pardonne, nous sommes chymiste, pas chirurgiens, il n'est pas à nous *d'operer.*

agreeable presage with regard to the ultimate recovery of her sight; both having a common origin, and, alike, symptoms of nervous debility or derangement.

One of the seven men who lately fell, as stated in the newspapers, from a scaffold in Thames-street, died upon the spot. In consequence of the horror which this accident excited in a near female relation, who was, before, in a weak and irritable state, she was seized with a paralytic affection of all her limbs. She is now a patient of the Dispensary.

This fact occurring only a few days after the one just stated, strongly co-operates with it, in demonstrating not the unlimited, but the important and incalculable, power which the mental faculties and sensations possess over the feelings and energies of the body.

In addition to the two instances already mentioned, tending to display the ascendancy of the mind over our material organization, the Reporter has a third case to relate, which likewise came under his cognizance, a very short time ago, in which a derangement of the body produced a corresponding disorder in the power and perspicacity of the understanding. It occurred at one of those critical periods of life, at which the female sex are particularly liable to an anomalous variety of diseases, especially to those to which there is any hereditary or constitutional propensity.

The poor woman fancied that she saw her bed encompassed with a legion of devils, impatient to hurry her to eternal torments. She derided medicine, and obstinately and haughtily resisted its application. In a very short time, however, an alteration having taken place in her physical condition, she repented of her folly, and smiled at the mention of her former terrors.

To so humiliating a degree do the floating particles of matter which surround, and still more those which enter into, the interior composition of our frame, exhibit their influence in exciting, repressing, or disordering the phenomena of human intelligence.

"Toi qui dans ta folie prends arrogamment le titre de *Roi de la Nature*; toi qui mesures et la terre et les cieux; toi, pour qui ta vanité s'imagine que le tout a été fait, parce que tu es intelligent, il ne faut qu'un léger accident, qu'un atome déplacée, pour te faire perir, pour te dégrader, pour te ravir cette intelligence, dont tu parois si fier."

*Greville Street, Brunswick Square, June* 21, 1805. J. REID.

